# PDX-1 Is a Therapeutic Target for Pancreatic Cancer, Insulinoma and Islet Neoplasia Using a Novel RNA Interference Platform

**DOI:** 10.1371/journal.pone.0040452

**Published:** 2012-08-08

**Authors:** Shi-He Liu, Donald D. Rao, John Nemunaitis, Neil Senzer, Guisheng Zhou, David Dawson, Marie-Claude Gingras, Zhaohui Wang, Richard Gibbs, Michael Norman, Nancy S. Templeton, Francesco J. DeMayo, Bert O'Malley, Robbi Sanchez, William E. Fisher, F. Charles Brunicardi

**Affiliations:** 1 Department of Surgery, David Geffen School of Medicine at University of California Los Angeles, Los Angeles, California, United States of America; 2 Department of Pathology, David Geffen School of Medicine at University of California Los Angeles, Los Angeles, California, United States of America; 3 Michael E. DeBakey Department of Surgery, Baylor College of Medicine, Houston, Texas, United States of America; 4 Elkins Pancreas Center, Baylor College of Medicine, Houston, Texas, United States of America; 5 Department of Molecular and Cellular Biology, Baylor College of Medicine, Houston, Texas, United States of America; 6 Human Genome Sequencing Center, Baylor College of Medicine, Houston, Texas, United States of America; 7 Gradalis, Inc., Dallas, Texas, United States of America; 8 Mary Crowley Cancer Research Centers, Dallas, Texas, United States of America; 9 Texas Oncology, P.A., Dallas, Texas, United States of America; Beckman Research Institute of the City of Hope, United States of America

## Abstract

Pancreatic and duodenal homeobox-1 (PDX-1) is a transcription factor that regulates insulin expression and islet maintenance in the adult pancreas. Our recent studies demonstrate that PDX-1 is an oncogene for pancreatic cancer and is overexpressed in pancreatic cancer. The purpose of this study was to demonstrate that PDX-1 is a therapeutic target for both hormonal symptoms and tumor volume in mouse models of pancreatic cancer, insulinoma and islet neoplasia. Immunohistochemistry of human pancreatic and islet neoplasia specimens revealed marked PDX-1 overexpression, suggesting PDX-1 as a “drugable” target within these diseases. To do so, a novel RNA interference effector platform, bifunctional shRNA^PDX-1^, was developed and studied in mouse and human cell lines as well as in mouse models of pancreatic cancer, insulinoma and islet neoplasia. Systemic delivery of bi-shRNA^humanPDX-1^ lipoplexes resulted in marked reduction of tumor volume and improved survival in a human pancreatic cancer xenograft mouse model. bi-shRNA^mousePDX-1^ lipoplexes prevented death from hyperinsulinemia and hypoglycemia in an insulinoma mouse model. shRNA^mousePDX-1^ lipoplexes reversed hyperinsulinemia and hypoglycemia in an immune-competent mouse model of islet neoplasia. PDX-1 was overexpressed in pancreatic neuroendocrine tumors and nesidioblastosis. These data demonstrate that PDX-1 RNAi therapy controls hormonal symptoms and tumor volume in mouse models of pancreatic cancer, insulinoma and islet neoplasia, therefore, PDX-1 is a potential therapeutic target for these pancreatic diseases.

## Introduction

Pancreatic and duodenal homeobox-1 (PDX-1) is a transcription factor that plays a critical role in regulating embryologic pancreas development as well as in insulin expression and islet maintenance in the adult pancreas [1], [2], [3], [4]. PDX-1 knockout is lethal in mice and mutation of PDX-1 leads to mature onset of diabetes of the young (MODY subtype IV) in mice and human patients [5], [6]. Persistent overexpression of PDX-1 leads to acinar to ductal cell metaplasia in mice [7]. Overexpression of PDX-1 in cell lines results in transformation of non-insulin-producing cells into insulin-producing cells upon GLP-1 stimulus [8], [9], [10], [11], [12], [13]. PDX-1 is well known as an essential regulator of many pancreatic endocrine genes such as insulin [1], [2], [3], [4], glucokinase [14], islet amyloid polypeptide [15], [16], [17], glucose transporter type 2 [GLUT2] [18], [19], pancreatic polypeptide [20] and somatostatin [21], [22], and therefore has a critical role in maintaining glucose homeostasis.

Our recent studies demonstrate that PDX-1 is an oncogene [23], [24]. It is markedly overexpressed in pancreatic cancer [23], [25], [26], [27] and regulates proliferation and invasion of human pancreatic cancer cells *in vitro* and *in vivo* in mice [23]. PDX-1 overexpression in benign and malignant cells resulted in increased tumor formation when these cells are implanted in mice [24]. Systemic delivery of shRNA^humanPDX-1^ lipoplexes resulted in a marked reduction of tumor volume and prolonged survival in a human pancreatic cancer xenograft mouse model [23].

Our team has developed a novel bifunctional RNA interference platform in which translation of the target mRNA is repressed via both cleavage-independent and cleavage-dependent RISC loading pathways, resulting in differential Argonaut incorporation and separate, but coordinated, target inactivation mechanisms [28].

In this study, a novel RNAi effector platform targeting PDX-1 was developed and studied in human and mouse cell lines, as well as in mouse models of pancreatic cancer, insulinoma and islet neoplasia to determine whether PDX-1 is a potential therapeutic target for control of both hormonal symptoms and tumor volume in these pancreatic diseases.

## Materials and Methods

### Ethics Statement

SCID mice were housed in a BL-4 facility and cared for in compliance with the guidelines in *The Care and Use of Laboratory Animals* prepared by the Institute of Laboratory Animal Resources, the Commission on Life Science, the National Research Council and the Animal Research Committee of Baylor College of Medicine. The protocol was approved by the Committee on the Ethics of Animal Experiments of the Baylor College of Medicine (Permit Number: AN 2404).

Human specimens were received under a human protocol that was approved by the Institutional Review Board (IRB) of Baylor College of Medicine (Permit Number: H 14054); specimens were processed strictly according to the procedures dictated by said protocol. All human pancreatic neuroendocrine tumor and nesidioblastosis samples were de-identified samples obtained with IRB consent (H 14054) and informed written consent.

### Cell lines, vectors, and antibodies

Mouse βTC-6 and human pancreatic cancer cell line PANC-1 cell lines were obtained from the American Type Culture Collection (ATCC, Bethesda, MD) and previously reported [29]. Human pancreatic neuroendocrine tumor specimens and human nesidioblastosis samples were kindly provided by Dr. David Dawson of the Department of Pathology at UCLA and Dr. Milton Finegold of the Department of Pathology at Texas Children's Hospital, respectively. Mouse PDX-1 shRNA (shRNA^mousePDX-1^) was designed as previously described [23]. Bi-functional mouse (bi-shRNA^mousePDX-1^) and human PDX-1 (bi-shRNA^humanPDX-1^) were obtained from Gradalis Inc (Dallas, TX). pRIP-mCherry_CMV-eGFP and pRIP-mCherry _CMVeGFP_H1-shRNA^mousePDX-1^ were subcloned based on the parent vector RIP-mCherry-NLS-mCherry that was provided by Dr. Michael Mancini from Baylor College of Medicine. Goat anti-rabbit anti-serum and sheep anti-mouse anti-serum conjugated with horseradish peroxidase were purchased from Amersham (Amersham Life Science Inc., Arlington Heights, IL). Rabbit anti-goat IgG was obtained from ZYMED (Zymed Laboratories, Inc. South San Francisco, CA, USA).

### Transfection and reporter assay

β TC-6 or PANC-1 cells were plated into 10-cm cell culture plates at 1×10^6^ cells per dish or 24-well plates at 1×10^5^ cells/well and incubated at 37°C for 24 h. Transfection assays were performed using Lipofectamine 2000 (Invitrogen, Carlsbad, CA, USA) according to manufacturer's instruction. Fluorescence signals were observed and counted using fluorescence microscopy as previously described [23] to determine the reporter activities.

### Cell proliferation assay *in vitro*

Cell proliferation was determined by MTS assay (Promega, Madison, WI) and BrdU incorporate assay (colorimetric) (Roche Diagnosistics GmbH, Mannherim, Germany) at 12, 24, 48 and 72 h post transfection. Absorbance was read in a Multiskan EX plate reader (Thermo Electronic Corp., Franklin, MA) at 492 nm for MTS and 500 nm for BrdU assay and levels of proliferation were calculated as described previously.

### Western blot analyses

Forty-eight hours after transfection, as described previously [23], βTC-6 cells were collected and lysed in RIPA buffer. Twenty (20) µg of cell lysates were applied to SDS-PAGE gel for electrophoresis. The protein was then transferred to membranes to be probed with various antibodies against PDX-1, cyclin D1, cyclin E, Cdk2, Cdk4, and p27. Immunocomplexes were visualized by enhanced chemiluminescence (ECL) detection using horseradish peroxidase conjugated secondary antibodies.

### Animals and shRNA delivery

SCID mice were housed in a BL-4 facility and cared for in compliance with the guidelines in *The Care and Use of Laboratory Animals* prepared by the Institute of Laboratory Animal Resources, the Commission on Life Science, the National Research Council and the Animal Research Committee of Baylor College of Medicine. DNA dosages were determined by meeting the criteria of less than 10% mouse death after injection of liposomal shRNA complex in regular SCID mice. Male mice, aged 8- to 10-weeks-old, were inoculated with 1×10^6^ β TC-6 or PANC-1 cells per mouse via intraperitoneal (ip) injection. Two weeks later, either β TC-6 or PANC-1 mice were randomly grouped (30 mice per group) and the first cycle of shRNA^mousePDX-1^ or bi-shRNA^mousePDX-1^ or bi-shRNA^humanPDX-1^, respectively, were given via tail vein injection. The cycles were repeated at days 14 and 28 for a total of three injections. The same protocol was applied to SSTR*1/5* ^−/−^ mice using 3 cycles of 35 µg of shRNA^mousePDX-1^. The shRNA lipoplexes were prepared as previously described [23].

### Insulin and glucose measurements

On days 7, 21, 35, and 120 following each gene delivery, 50 µl whole blood samples were collected and spun to separate the serum. Glucose levels were measured using a Beckman-Coulter Glucose Analyzer 2 (Coulter-Beckman, Fullerton, CA), and presented as mean ± S.E.M. in mg/dl. Insulin levels were determined using a mouse insulin ELISA kit from Mercodia (Linco Research, St. Charles, MO) and presented as mean ± S.E.M. in µg/l.

### Intraperitoneal Glucose Tolerance Test (IPGTT)

SSTR*1/5* ^−/−^ mice at days 7 and 150 after initial delivery of shRNA^mousePDX-1^ were fasted overnight before collection of blood samples as T_0_. Grouped mice were then given 1.2 g glucose/kg body weight via ip injection followed by collection of blood samples at 15, 30, 60, and 120 min after injection of glucose. Glucose and insulin levels were measured as described above.

### Necropsy, tissue collection, immunohistochemistry and TUNEL assay

De-identified human pancreatic neuroendocrine tumor and nesidioblastosis samples were obtained from Dr. David Dawson and from Dr. William Fisher for IHC. Mouse pancreas, islets and tumor tissue samples were obtained on days 0, 7, 21, 35, and end time after initial gene delivery. Peritoneal tumors were macroscopically and microscopically evaluated and the larger (A) and smaller (B) diameters measured and recorded. Tumor volume (V; a rotational ellipsoid) was calculated according to the formula: V (mm^3^) = A(mm)×B^2^(mm)^2^/2. Total tumor volume was calculated by putting all tumors together. Mice were scored according to presence or absence of tumor. IHC was performed as described previously. Anti- PDX-1, insulin, PP, cyclin E, p27, Cdk4 or PCNA antibodies were applied to slides with 1∶100 dilution followed by overnight incubation at 4°C. Slides were incubated with Cy3 or FITC-conjugated anti-rabbit, goat, or mouse secondary antibody depending on derivation of primary antibodies for one hour, and mounted with cover slides. TUNEL assay (FragEL™ DNA Fragmentation Detection Kit, Colorimetric-TdT Enzyme, CALBIOCHEM, La Jolla, CA) was performed according to the manufacturer's protocol. The rate of apoptosis was expressed as the ratio of apoptotic cancer cells to the total number of endothelial cells in 10 fields at ×100 magnification.

### Statistical Analysis

The unpaired Student's *t*-test was used for statistical analyses of glucose levels, insulin levels, and cell proliferation with *P*<0.05 indicating significance. The χ^2^ test was used for rate comparison. Rank-log was used for mice survival comparison. Kaplan-Meier in SPSS 15.0 for MS Windows was used to plot survival curves.

## Results

### PDX-1 is overexpressed in human specimens of pancreatic neuroendocrine tumors and nesidioblastosis, as well as mouse insulinoma cells and a transgenic islet hyperplasia mouse model

**Figure 1:**
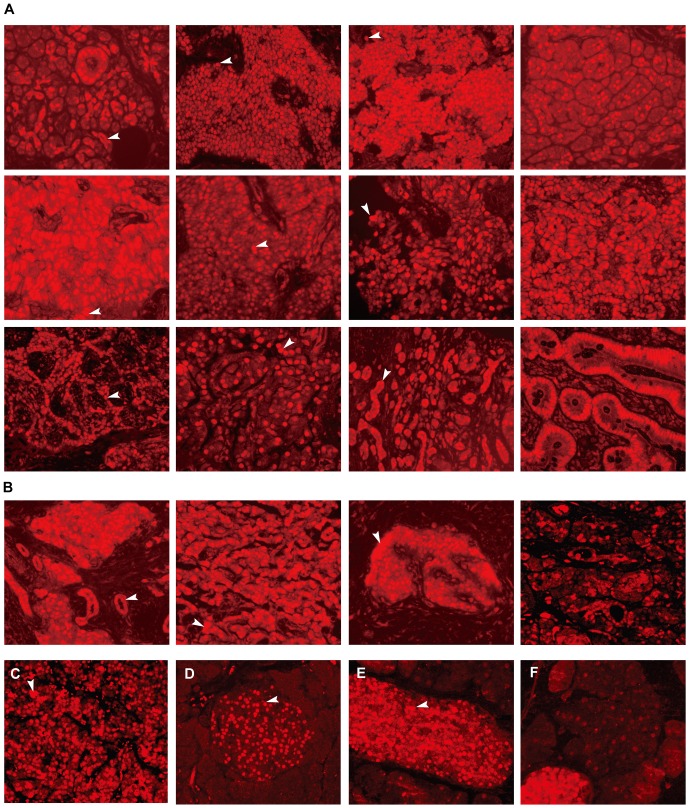
PDX-1 expression in islet neoplasia, β TC-6 tumor and islet of SSTR*1/5* ^−/−^. Immunostaining with anti-PDX-1 antibody demonstrates overexpression of PDX-1 in human islet neoplasia specimens (26 pancreatic neuroendocrine tumors (a), 10 nesidioblastosis specimens (b), βTC-6 tumors (c) and 6 pancreata specimens from SSTR1/5^−/−^ mice (e, f). PDX-1 is overexpressed in both islets (e) and acinar cells (f) of SSTR1/5^−/−^ mice as compared to that of wild type mice where only islet cells expressed PDX-1 (d). PDX-1 positive is indicated by arrow (×200).

PDX-1 was overexpressed in 36 human pancreatic and islet neoplasia specimens including 26 pancreatic neuroendocrine tumors (Fig. 1a) and 10 nesidioblastosis specimens (Fig. 1b). PDX-1 also was overexpressed in mouse insulinoma (β TC-6) cells (Fig. 1c) and in both islet and acinar cells of the pancreata of somatostatin receptor subtypes 1 and 5 knock-out mice (SSTR*1/5* ^−/−^) (Fig. 1e, f, respectively) as compared to that of wild type mice (Fig. 1d). These data demonstrate that PDX-1 is significantly overexpressed in both human pancreatic neuroendocrine tumors, nesiodioblastosis and mouse islet neoplasia, suggesting it is a potential target for these diseases.

### Targeting PDX-1 with bi-shRNA^humanPDX-1^ controls tumor volume in a human pancreatic cancer xenograft mouse model

#### *In vitro* studies

**Figure 2:**
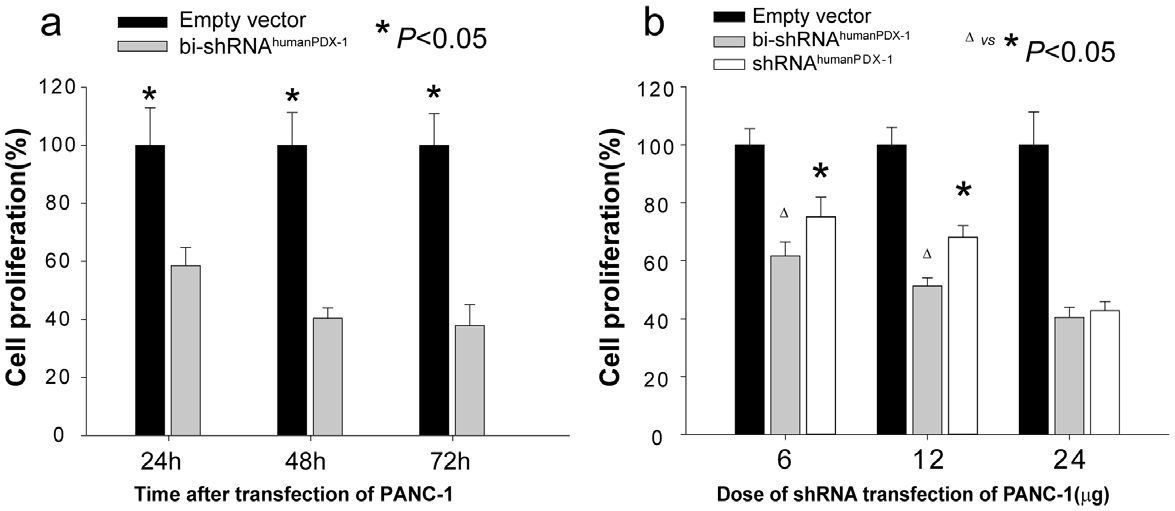
Knockdown of PDX-1 expression inhibits cell proliferation in PANC-1 cells. MTS assay showed the time course of cell proliferation for PANC-1 (a) cells transfected with bi-shRNA^mousePDX-1^ and bi-shRNA^humanPDX-1^, respectively. A comparison of cell proliferation inhibition percentages between bi-shRNA and shRNA transfection at different doses is shown in PANC-1 cells (b).

Since PANC-1 cells are human pancreatic cancer cells that markedly overexpress PDX-1, these cells were used for both *in vitro* and *in vivo* studies. bi-shRNA^humanPDX-1^ transfection of PANC-1 cells resulted in knock down of PDX-1. This was associated with reduction of cell proliferation by 58%, 40% and 38% compared to empty vector controls at 24, 48 and 72 h post-transfection, respectively. There was greater inhibition of cell proliferation than that seen with shRNA^humanPDX-1^ at the 0.6 ug and 1.2 ug/ml medium doses (Fig. 2a, 2b), addressing the potential advantage of bi- shRNA^humanPDX-1^ in the RNAi therapy for pancreatic cancers.

#### *In vivo* studies

**Figure 3:**
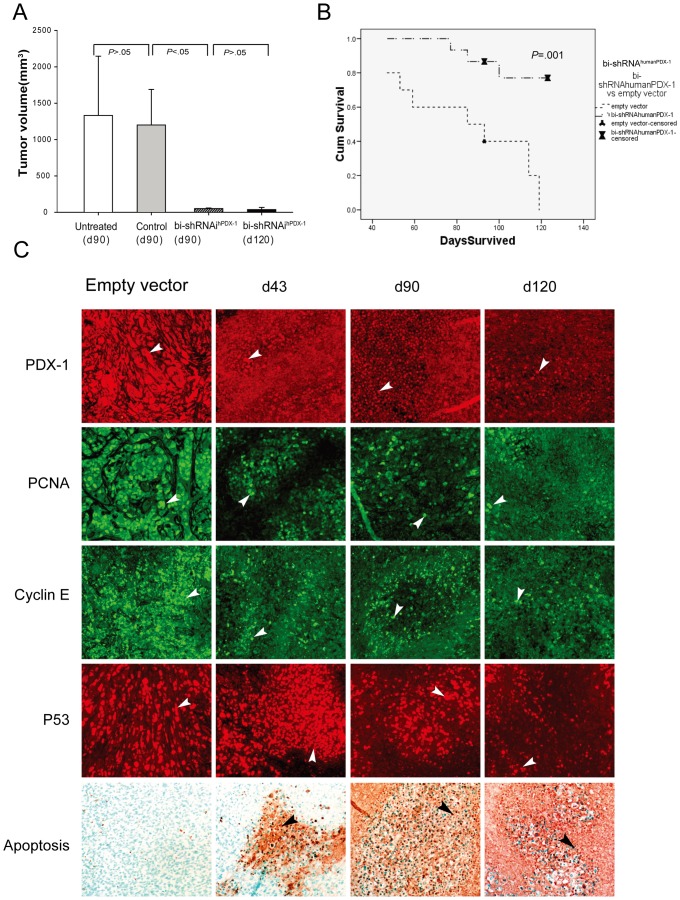
shRNA^humanPDX-1^ therapies reduce tumor volume and prolong survival in a xenograft SCID mouse model of pancreatic cancer. The tumor volume was evaluated and compared at 90 and 120 days following the initial treatment between treatment and control groups (a). The survival rates of mice receiving treatment and of those receiving the empty vector control were analyzed using Kaplan-Meier in SPSS (b). Immunostaining for tumor slides with PDX-1, PCNA, Cyclin E, and P53 as well as TUNEL assay were performed and analyzed (c). The positive for each marker is indicated by arrow (×200).

A PANC-1 xenograft SCID mouse model has been developed and well studied in our laboratory; when placed intraperitoneally in SCID mice, PANC-1 cells grew into large tumors within two months. Two weeks following implantation of the cells, three cycles of bi-shRNA^humanPDX-1^ lipoplexes (35 µg/mouse, intravenously administered at days zero,14 and 28) significantly reduced PANC-1 tumor volume with only a few mice having residual tumors averaging 51 mm^3^ compared to 100% of control mice with tumors averaging 1199 mm^3^ at 90 days post-treatment (p<0.05; Fig. 3a). Survival in the treatment group was significantly longer than controls (115±9.5d vs 85±1.4d, respectively; (p = 0.001; Fig. 3b) with no significant difference in survival between empty vector-treated mice and untreated mice (85±9d vs 76±8d, p = 0.981). In those mice with residual tumors, tumor PDX-1 expression was significantly reduced from 95±11.1% to 12±1.3% on days 0 and 90 post-treatment, respectively (Fig. 3c top panel). IHC analysis of residual tumors revealed decreased expression of cell proliferation markers, proliferative cell nuclear antigen (PCNA) and Cyclin E, (from 84±9.6% to 15±0.8% and from 66±10.2% to 9±2.3%, respectively; Fig. 3c 2^nd^ and 3^rd^ panels), and an increase in P53 expression (Fig. 3c 4^th^ panel). Marked apoptosis was seen in residual tumors at day 90 post-treatment, suggesting the residual tumors were composed of apoptotic cells that lack PDX-1 expression. (Fig. 3c bottom panel). Remarkably, three cycles of bi-shRNA^humanPDX-1^ lipoplexes had no effect on systemic glucose and insulin levels, underscoring the importance of the species-specific design of PDX-1 RNAi (Fig. S1).

These data demonstrate that multiple cycles of systemic bi-shRNA^humanPDX-1^ lipoplexes were well tolerated and effectively reduced human pancreatic cancer tumor volume and prolonged survival in SCID mice. These data demonstrate that PDX-1 is a potential target using this novel therapeutic platform for the most aggressive form of pancreatic neoplasia.

### Targeting PDX-1 with bi-shRNA^mousePDX-1^ controls excessive secretion of insulin from mouse insulinoma cells and in a mouse model of insulinoma

#### *In vitro* studies

**Figure 4:**
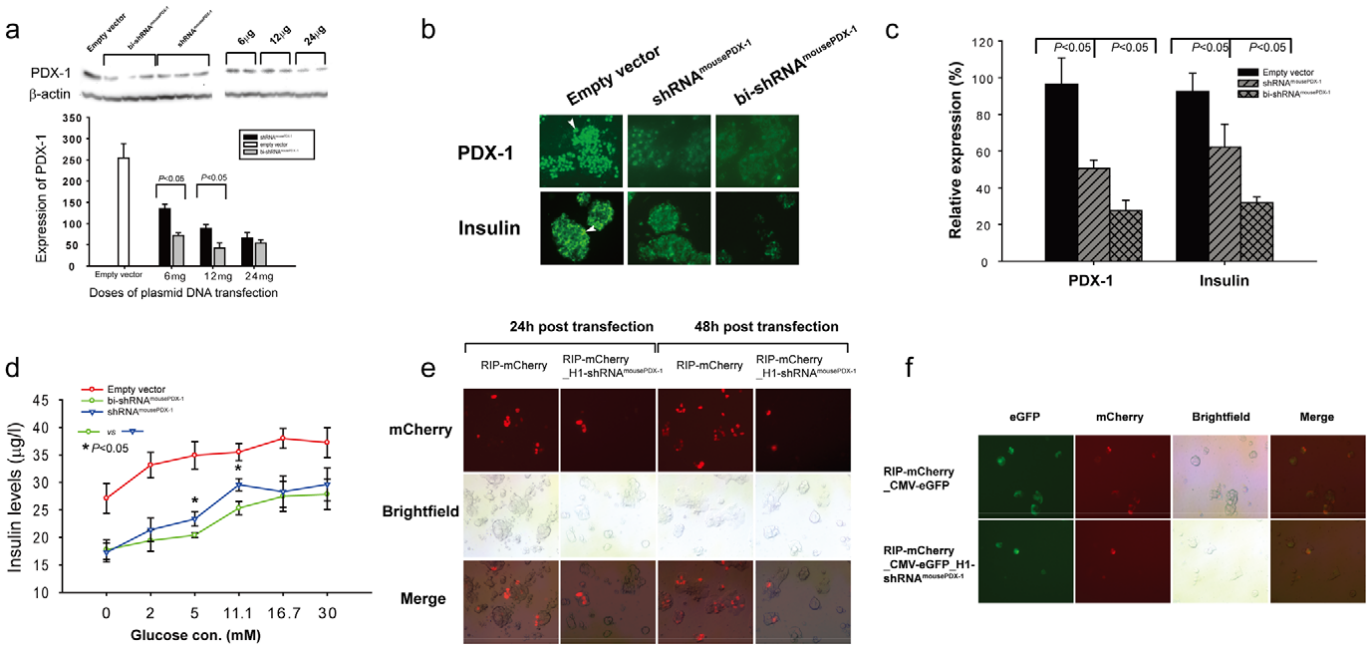
bi-shRNA^mousePDX-1^ knockdown of mPDX-1 expression in β TC-6 cells *in vitro* inhibits insulin expression and secretion. Comparison of efficacy of bi-shRNA^mousePDX-1^, shRNA^mousePDX-1^ or empty vector in inhibition of PDX-1 expression in β TC-6 cells is shown using western blot (a) and cell immunostaining (top panel, b as indicated by arrow). Insulin expression and glucose stimulated secretion in response to knockdown of PDX-1 is shown by cell immunostaining (bottom panel, b as indicated by arrow) (×200) and ELISA assay (d), respectively. Each experiment was repeated five times. PDX-1 and insulin expression in immunostained β TC-6 cells are quantified (c). PDX-1 expression affected the RIP-directed reporter expression (RIP-mCherry) in βTC-6 cells (e and f). The cells expressing mCherry (red) and GFP (green) were visualized and photographed using fluorescence microscopy (e and f). (×200).

bi-shRNA^mousePDX-1^ resulted in significantly greater knockdown of PDX-1 expression in β TC-6 cells at doses of 0.6 µg and 1.2 µg/ml medium than shRNA^mousePDX-1^ and empty vector controls in both western blot (Fig. 4a; p<0.05) and immunohistochemistry (IHC) analyses (Fig. 4b top panel).

bi-shRNA^mousePDX-1^ resulted in significantly greater inhibition of insulin expression and PDX-1 expression than that seen with shRNA^mousePDX-1^ and empty vector control cells, respectively (Fig. 4c, p<0.05; and Fig. 4b). Transfection with bi-shRNA^mousePDX-1^ resulted in significantly greater inhibition of glucose-stimulated insulin secretion from β TC-6 cells *in vitro* compared to that seen with shRNA^mousePDX-1^ and empty vector controls at glucose concentrations of 5 and 11 mM (p<0.05), respectively (Fig. 4d). These findings indicate that bi-shRNA^mousePDX-1^ inhibits insulin expression and secretion via PDX-1 knockdown, and do so to a greater degree than conventional shRNA^mousePDX-1^ in mouse insulinoma cells.

To further delineate the mechanism by which knockdown of PDX-1 inhibits insulin expression and secretion through reduced activation of rat insulin promoter-1(RIP), reporter constructs (RIP-mCherry) with or without shRNA^mousePDX-1^ were transfected into β TC-6 cells. RIP-mCherry-H1-shRNA^mousePDX-1^ resulted in significant reduction in mCherry expression as compared to that of RIP-mCherry without shRNA^mousePDX-1^ (10±1.6% vs 35±8.1% and 12±2.1% vs 66±13.2% at 24 h and 48 h, respectively, p<0.05; Fig. 4e). Transfection of RIP-mCherry-CMV-eGFP-H1 revealed that 90% of cells were successfully transfected, as evidenced by eGFP expression. Additionally, 69% of eGFP-expressing cells expressed mCherry, and in RIP-mCherry-CMV-eGFP-H1-shRNA^mousePDX-1^ transfected cells, 88% of cells expressed eGFP; however, only 12% of cells expressed mCherry (p<0.05; Fig. 4f). Decreased PDX-1 expression was confirmed using western blot. These data suggest that activation of the insulin promoter is significantly inhibited by shRNA^mousePDX-1^ via knockdown of PDX-1 expression.

**Figure 5:**
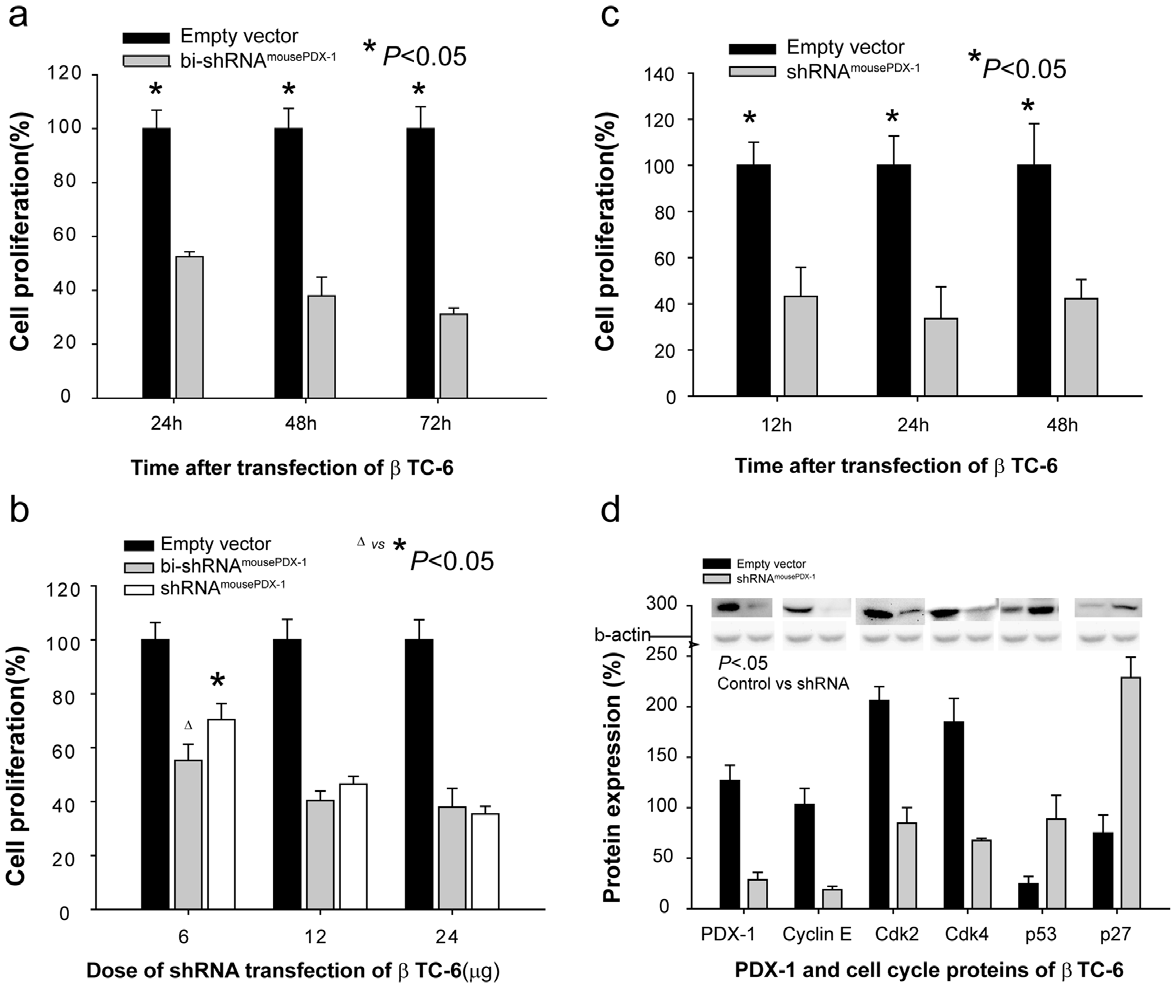
Knockdown of PDX-1 expression inhibits cell proliferation in β TC-6 cells. MTS assay showed the time course of cell proliferation for β TC-6 (a) cells transfected with bi-shRNA^mousePDX-1^ and bi-shRNA^humanPDX-1^, respectively. A comparison of the percentages of cell proliferation inhibition between bi-shRNA and shRNA transfection at different doses is shown in β TC-6 cells (b). Cell proliferation determined by BrdU also is shown (c). Western blot analysis of 20 ug lysate from β TC-6 cells that were transfected with shRNAi^mousePDX-1^ after 48 h was performed with anti-PDX-1, cyclin E, Cdk2, Cdk4, p53 and p27 (d).

Transfection with bi-shRNA^mousePDX-1^ into β TC-6 cells resulted in significant suppression of cell proliferation using two different assay: the MTS assay revealed reductions of 52%, 38%, and 31% versus empty vector controls at 24, 48, and 72 h post-transfection, respectively (Fig. 5a), and the BrdU incorporation assay revealed reductions of 42%, 35%, and 42% at 12, 24, and 48 h post-transfection, respectively, (Fig. 5b) (p<0.05 at all time points). bi-shRNA^mousePDX-1^ resulted in greater inhibition of cell proliferation than shRNA^mousePDX-1^ at low doses (6 ug/10-cm dish) after 48 h of transfection (Fig. 5c). Cell cycle protein analysis demonstrated greater down-regulation of positive regulators, cyclin E, Cdk2, and Cdk4, and up-regulation of negative regulators, p53 and p27, in bi-shRNA^mousePDX-1^-treated cells compared to that of empty vector controls (p<0.05; Fig. 5d). These data demonstrate that PDX-1 knockdown inhibits mouse insulinoma cell proliferation via alterations in cell cycle proteins.

#### *In vivo* studies

**Figure 6:**
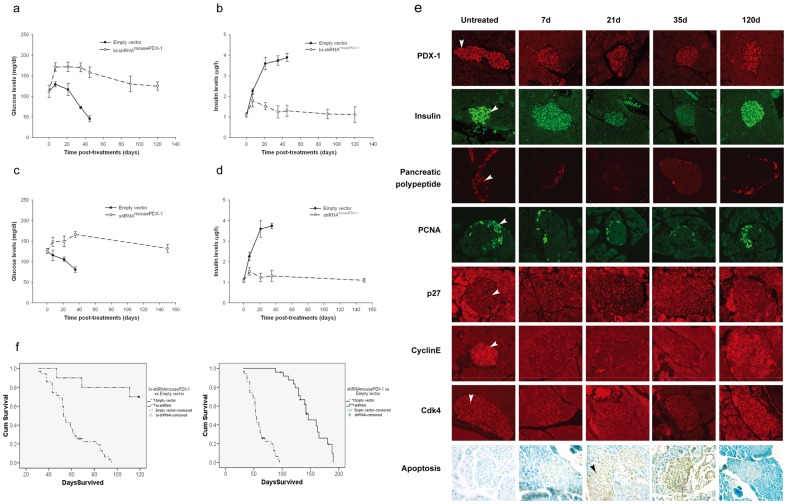
Systemic bi-shRNA^mousePDX-1^ lipoplexes prevent death from severe hyperinsulinemia and hypoglycemia in an insulinoma SCID mouse model. Glucose levels A and C corresponding to insulin levels B and D were acquired from the β TC-6 mice treated with bi-shRNAi^mousePDX-1^ or shRNAi^mousePDX-1^, respectively. In each figure a-d, the dash line represents control group data and the dash-dot line represents treatment group data. Islet cells expressing PDX-1, insulin, PP, PCNA, p27, cyclin E and CdK4 were shown by IHC and islet cell apoptosis was shown by TUNEL assay, as indicated by arrows (×200) (e). Mouse survival after three treatment cycles of either empty vector or bi-shRNAi^mousePDX-1^ and empty vector or shRNAi^mousePDX-1^ was evaluated and compared using Kaplan-Meier in SPSS (f).

Previously, our laboratory developed a lethal mouse insulinoma model, which has been well studied. SCID mice are implanted with mouse insulinoma cells and succumb to hyperinsulinemia and hypoglycemia at approximately 60 days. In this mouse model, three cycles of either intravenous bi-shRNA^mousePDX-1^ lipoplexes (25 ug/mouse biweekly) or shRNA^mousePDX-1^ lipoplexes (35 ug/mouse biweekly) prevented death from hyperinsulinemia and hypoglycemia (Fig. 6a and b, and c and d, respectively). Transient hyperglycemia and hypoinsulinemia were observed, but levels returned to baseline on day 150 after the initial treatment in both treatment groups. In the control group, empty vector lipoplexes had no effect on lethal hyperinsulinemia and hypoglycemia, as shown in Fig. 6a and b.

Overall survival in the bi-shRNA^mousePDX-1^ and shRNA^mousePDX-1^ treatment groups was significantly longer than that of controls (106±7.8d vs 53±1.4d and 146±9.5d vs 53±1.4d, respectively, p<0.05 vs controls, Fig. 6f, Fig. S2). It is to be noted that the insignificant difference in survival between the two treatment groups was due to intentional sacrificing of the bi-shRNA^mousePDX-1^ group of mice at an earlier time point (Fig. S2f). These data demonstrate that PDX-1 knockdown using systemic bi-shRNA^mousePDX-1^ lipoplexes effectively prevents hyperinsulinemic, hypoglycemic death in an insulinoma SCID mouse model; therefore, the treatments control excessive hormonal secretion associated with this form of islet neoplasia.

Remarkably, systemic bi-shRNA^mousePDX-1^ and shRNA^mousePDX-1^ lipoplexes result in temporal hyperglycemia, representing a predictable off-target effect on mouse islets, since PDX-1 is the predominant regulator of insulin expression. After treatment with bi-shRNA^mousePDX-1^, *in situ* islet PDX-1 expression was reduced in a treatment-dependent manner, from 88±10.1% on day 0 to 71±8.6%, 49±9.7% and 23±6.6% on days 7, 21, 35 after the treatments, respectively, and returned to 83±12.4% on day 120 after treatments, as shown in the Fig. 6e, top panel. Insulin expression also was significantly decreased from 92±8.3% on day 0 to 61±9.8%, 32±5.3%, and 12±1.9% on days 7, 21, 35 after the treatments, respectively, and returned to 89±15.3% on day 120 after treatments, as shown in Fig. 6e, 2^nd^ panel. Expression levels of PP also decreased significantly at the same time points after treatment (Fig. 6e, 3^rd^ panel). Markers of islet cell proliferation, cell cycle proteins and apoptosis were also studied pre- and post-treatment. Islet PCNA expression significantly decreased from 16±1.4% to 10±0.8%, 7±0.6%, 5±0.2% and 14±2.0% on days 0, 7, 21, 35 and 120 after treatments, respectively (Fig. 6e, 4^th^ panel). Both IHC and western blot analyses of pancreatic sections demonstrated increasing p27 expression and decreasing expression of protein levels in cyclin E and Cdk4 following three cycles of treatment (Fig. 6e, 5^th^, 6^th^ and 7^th^ panels, and Fig. S3, respectively). bi-shRNA^mousePDX-1^ resulted in a significant increase in apoptosis of mouse islets from 1±0.2% on day 0, to 14±2.4%, 24±3.6%, 42±5.5%, and 10±1.1% on days 7, 21, 35 and 120 after treatments, respectively (Fig. 6e bottom panel). Empty vector control therapy had no effect on islet PDX-1, insulin, PP expression, cell cycle proteins and apoptosis. These data are consistent with findings seen following PDX-1 knockdown in mouse insulinoma cells *in vitro*. They also suggest that systemic bi-shRNA^PDX-1^ lipoplexes result in temporal, mild hyperglycemia emanating from suppression of PDX-1 within the islets of Langerhans with subsequent suppression of insulin and PP expression, correlating with alterations in islet cell cycle proteins and increased islet apoptosis.

Expression of all islet markers returned to baseline values upon sacrifice, suggesting a regenerative capacity of murine islets, resulting in normalization of basal insulin and glucose levels. This is in contrast to the PANC-1 cell residual tumors that had low levels of PDX-1 and markedly elevated apoptosis, suggesting a lack of regeneration. Similar patterns of expression of PDX-1, insulin, PP, and cell cycle proteins and apoptosis in islet cells also were observed after three cycles of shRNA^mousePDX-1^ lipoplexes (Fig. S4). These data demonstrate that systemic bi-shRNA^mousePDX-1^ lipoplexes effectively prevent hypoglycemic death in an insulinoma SCID mouse model and result in *in situ* knockdown of PDX-1 within the islets, leading to suppression of insulin expression and secretion and subsequent hyperglycemia. Remarkably, any off-target islet effects were mild and temporal, suggesting a regenerative capacity of the murine endocrine pancreas. Since there are no residual insulinoma cells found in this model after therapy, the islet data demonstrate the ability of the systemically-delivered treatment to knockdown PDX-1 in this model.

### Systemic shRNA^mousePDX-1^ lipoplexes reverse hyperinsulinemia and hypoglycemia and alter glucose tolerance in somatostatin receptor subtypes 1 and 5 (SSTR*1/5* ^−/−^) knockout mice

**Figure 7:**
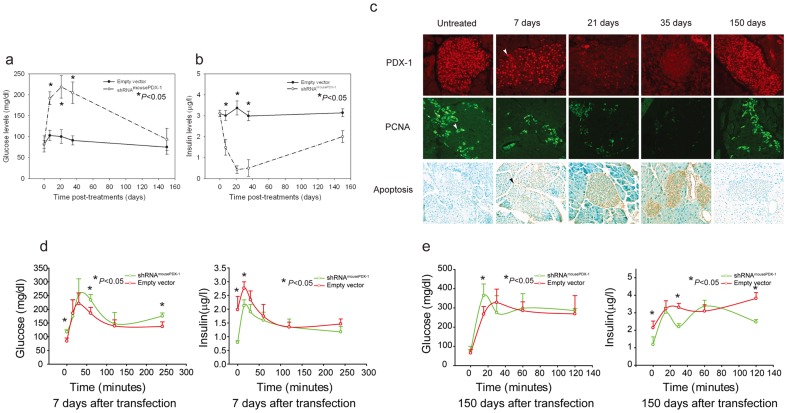
Systemic shRNA^mousePDX-1^ lipoplexes reverse hyperinsulinemia and hypoglycemia and alter glucose tolerance in somatostatin receptor subtypes 1 and 5 (SSTR1/5^−/−^) knockout mice. Glucose levels (a) and corresponding insulin levels (b) were measured as described in the method section. Immunostaining for pancreatic slides with PDX-1, PCNA and TUNEL assay were performed. Red staining in top panel of (c) indicates PDX-1 expression (arrow); green staining at middle panel (c) indicates PCNA expression (arrow). Apoptotic tumor cells from SSTR1/5^−/−^ mice are stained brown and are shown in the bottom panel of (c) (arrow) (×200). IPGTT assay showed glucose levels and insulin levels (d) on day 7 and glucose levels and insulin levels (e) on day 150 after shRNA^mousePDX-1^ therapy. Data are presented as means ± S.E.M. and P<0.05 indicate significance.

The SSTR1/5^−/−^ mouse model was used as the mice represent an immune-competent insulinoma-like mouse model with overexpression of PDX-1 in both acinar cells and islet cells, featured with hyperinsulinemia and hypoglycemia. We wanted to determine whether multiple intravenous treatments would reverse basal hyperinsulinemia and hypoglycemia and be tolerated in these immune-competent mice. Three cycles of shRNA^mousePDX-1^ lipoplexes (35 ug/mouse) were well tolerated and significantly reversed basal hyperinsulinemia and hypoglycemia in SSTR*1/5* ^−/−^ mice (3±0.2 ug/l and 83±19.5 mg/dl on day 0 and 0.5±0.1 ug/l and 205±25.9 mg/dl on day 35 after three treatments, respectively, as seen in Fig. 5a and b); empty vector lipoplexes had no effect on hyperinsulinemia and hypoglycemia (Fig. 7a and b). Remarkably, systemic glucose and insulin levels returned to baseline on day 150 after the initial treatment, suggesting a regenerative capacity of these murine islets.

Intraperitoneal glucose tolerance tests (IPGTT) were performed on SSTR*1/5* ^−/−^ mice at days 7 and 150 after the initial treatment to study glucose regulation in greater detail. Fasting glucose levels in the treatment group were significantly higher than those of controls one week post-treatment (117±6.8 mg/dl vs 84±5.9 mg/dl, p<0.05), whereas basal insulin levels in the treatment group were lower than those of controls (1±0.1 µg/l vs 2±0.2 µg/l, p<0.05; Fig. 7d left). Following intraperitoneal glucose injection, systemic glucose levels were significantly higher than those of controls at 60 and 240 min post-injection (234±20.2 mg/dl vs 189±21.0 mg/dl and 175±11.3 mg/dl vs 137±16.8 mg/dl, respectively p<0.05). Serum insulin levels were significantly lower than those of controls at 15 min post-injection (2±0.1 ug/l *vs* 3±0.2 µg/l) (Fig. 7d right). On post-treatment day 150, no significant difference in basal glucose levels was seen between treatment and control groups; however, basal insulin levels in the treatment group were significantly lower than in those of controls (1±0.3 µg/l vs 2±0.3 µg/l, p<0.05). Only the 15-minute time point demonstrated a significant difference in systemic glucose levels between the groups (364±60.1 vs 267.0±40.4, p<0.05) (Fig. 7e left). Systemic insulin levels; however, were significantly lower at the 30- and 120-minute time points in the treatment group as compared to controls, (2.2±0.3 ug/l vs 3.3±0.2 ug/l and 2.5±0.1 ug/l vs 3.8±0.3 ug/l at 30 and 120 min post-injection, respectively; p<0.05) (Fig. 7e right).

Upon sacrificing the mice, pancreata were fixed and prepared for immunohistochemistry. Islet PDX-1 expression was reduced in a treatment-dependent manner from 93±13.5% on day 0 to 12±2.6% on day 35 post-treatment; returning to baseline expression (88±15.2%) on day 150 post-treatment (Fig. 7c, top panel). Islet PCNA expression decreased significantly from 33±2.8 from day 0 to 6±0.6% on day 35 and returned to baseline (29±4.3%) on day 150 post-treatment, respectively (Fig. 7c middle panel). Similarly, islet expression of cyclin E, cyclin D, Cdk4 and Cdk2 was suppressed, and then returned to baseline on day 150. Progressive increases in islet cell apoptosis were observed (1±0.1%, 19±3.2%, 32±5.7%, 58±8.9% on days 0, 7, 14, and 35 post-treatment, then returned to baseline (9±1.4%) on day 150 (Fig. 7c bottom panel), suggesting regenerative capacity of these murine islets. No effect on expression of these proteins, or on islet apoptosis, was seen with empty vector controls.

These data demonstrate that three cycles of systemic shRNA^mousePDX-1^ lipoplexes were well tolerated and effective in reversing hyperinsulinemia and hypoglycemia in an immune-competent mouse model of pancreatic neoplasia. Significant alterations in glucose tolerance seven days after the initial treatment corresponded with suppressed islet PDX-1 and insulin expression and increased islet apoptosis. Only mild alterations in the response to IPGTT seen at 150 days post-treatment correspond with normalization of islet PDX-1, insulin expression, cell cycle proteins and apoptosis, and suggest a regenerative capacity of immune-competent murine islets.

## Discussion

This study demonstrates that targeting PDX-1 using a novel RNAi effector platform regulates both excessive hormonal secretion, as well as reduction of tumor volume, in mouse models of pancreatic cancer, insulinoma and islet neoplasia. Three systemic treatments were well tolerated in both immune-competent and immune-incompetent mice. There was a predictable off-target effect on islets, which was remarkably reversible, suggesting a regenerative capacity of the murine endocrine pancreas. However, small residual human pancreatic tumors had low expression of PDX-1 and marked apoptosis, suggesting the lack of a regenerative capacity of PANC-1 cells following treatment. These data, combined with an overexpression of PDX-1 seen in human specimens of pancreatic neuroendocrine tumors and nesidioblastosis, support the role of PDX-1 as a potential novel therapeutic target using this novel RNAi platform [28].

Our RNAi platform was effective in knocking down PDX-1 expression *in vitro* and *in vivo*. Although synthetic small interfering RNAs (siRNAs) are easier to deliver in preclinical investigations and have recently entered clinical studies, DNA cassettes that express small hairpin RNA (shRNA), microRNA (miRNA), or strands of siRNAs have the advantage of a prolonged effect [30], [31]. Transcribed shRNA from an expression vector intrinsically differs from siRNA with respect to intracellular trafficking and nucleotide preference and the bi-shRNA construct can further enhance gene knockdown [28]. Our novel bifunctional RNAi vector represses translation of the target mRNA via both cleavage-independent and cleavage-dependent RISC loading pathways, resulting in differential Argonaute incorporation and separate, but coordinated, target inactivation mechanisms [28]. The studies showed that both shRNA and bi-shRNA system were effectively working on the molecular target in vivo [23], [28].

In order to achieve multiple intravenous doses of the RNAi therapy, the liposomal delivery system used in this study was developed using 1,2-dioleoyl-3-trimethyl-ammoniopropane (DOTAP) and cholesterol bilamellar invaginated vesicle (BIV) [32]. The composition and structure of these lipoplexes result in an optimized approximate 5½ hour plasma half-life by minimizing interaction with plasma proteins and accumulation in non-target tissues; their fusogenic property avoids endocytosis, which otherwise can lead to nucleic acid degradation [33]. The safety of this lipoplex system to be administered intravenously in multiple cycles already has been demonstrated in other *in vivo* models against other targets [34], [35] and in clinical trials [36], [37].

In our xenograft mouse model of human pancreatic cancer, the most aggressive form of pancreatic neoplasia, our strategy of PDX-1 knockdown effectively reduced human pancreatic cancer tumor formation in SCID mice and prolonged survival [23]. It was remarkable that in the few mice with residual pancreatic cancer tumors, studies of those tumors revealed marked suppression of PDX-1 and marked enhancement of both P53 and apoptosis 90 days after therapy. This suggests that PDX-1 knockdown activated the apoptotic pathway via P53 within PANC-1 cells. These findings support the concept that PDX-1 has oncogenic properties and is a potential therapeutic target for pancreatic cancer. We previously reported that 80 human pancreas tissue specimens including liver metastases, had marked overexpression of PDX-1 [23], [24]. Together, these findings support the development of a phase I clinical trial using this novel RNAi platform targeting PDX-1 for treating this devastating pancreatic disease. Interestingly, there was no adverse effect of bi-shRNA^humanPDX-1^ on mouse islet PDX-1 nor insulin expression, as well as insulin and glucose levels, demonstrating the importance of species-specificity of the PDX-1 shRNA design.

Insulinoma is an intermediate form of islet neoplasia in which tumor formation occurs with a ratio of approximately 90% being benign, for which the treatment is surgical excision, and 10% being malignant, for which there is no effective treatment. However, the predominant symptom associated with this disease is intolerable hypoglycemia for which there is no effective treatment. The effect of PDX-1 knockdown using bi-shRNA^mousePDX-1^ on mouse insulinoma cells and mouse insulinoma model revealed 1) prevention of lethal hypoglycemia in our insulinoma SCID mouse model with mild, temporal hyperglycemia; 2) temporal suppression of islet PDX-1 and insulin expression and increased islet apoptosis, all of which reversed by day 150; 3) deactivation of an exogenous rat insulin promoter fragment; 4) decreased activation of endogenous insulin promoter resulting in inhibition of expression and secretion of insulin and 5) inhibition of cell proliferation via alterations in cell cycle proteins. It is to be noted that due to the rapid occurrence of malignant hypoglycemic death in this model, there is insufficient time for implanted β TC-6 insulinoma cells to develop measurable tumors before insulinoma-induced hypoglycemic death. Therefore, there is no tumor volume to be measured in this model. However, the development of temporal, mild hyperglycemia post-therapy associated with suppression of *in situ* islet PDX-1, insulin and PP, both known to regulate glucose homeostasis [15], [16], [17] and increased islet apoptosis, demonstrate that systemic delivery of bi-shRNA^mousePDX-1^ lipoplexes are reaching their PDX-1 target.

The survival advantage following multiple systemic cycles of bi-shRNA^mousePDX-1^ lipoplexes suggests that temporal, mild hyperglycemia might be an acceptable off-target toxicity in exchange for control of devastating hormonal symptoms, as well as anti-neoplasia activity [1], [2], [3], [4].

Combined with the finding that 36 human pancreatic neuroendocrine tumors and nesidioblastosis specimens had marked overexpression of PDX-1, these data suggest that our novel RNAi effector platform has clinical potential for treating both the hormonal symptoms associated with excessive hormonal secretion, as well as control of tumor volume associated with pancreatic neuroendocrine tumors [38]. This is important since there are no current effective medical treatments for the devastating hormonal symptoms associated with pancreatic neuroendocrine tumors and nesidioblastosis [38].

We chose to study the effect of shRNA^mousePDX-1^ in the SSTR*1/5* ^−/−^ mouse model to determine whether multiple treatments would be tolerated in an immune-competent mouse model of islet neoplasia [39], [40], [41]. Multiple treatments of intravenous shRNA^mousePDX-1^ lipoplexes were well tolerated and reversed hyperinsulinemia and hypoglycemia. Similar to the results seen in the insulinoma SCID mouse model, SSTR1/*5* ^−/−^ mice developed temporal hyperglycemia associated with elevated basal glucose levels, lower insulin levels and an abnormal response to IPGTT seven days after the first treatment. Examination of the islets revealed that the most likely mechanism was suppression of PDX-1, insulin and pancreatic polypeptide expression, disruption of cell cycle proteins, and increased islet apoptosis. Remarkably, these mice have an almost complete restoration of their response to glucose associated with the normalization of islet PDX-1, insulin, pancreatic polypeptide, and PCNA expression, as well as absence of islet apoptosis, suggesting a regenerative capacity of the murine endocrine pancreas in this immune-competent mouse model of islet neoplasia [39], [40], [41].

In summary, PDX-1, which exhibits both oncogenic and hormonal-regulatory properties, is a therapeutic target in three mouse models of progressive pancreatic and islet neoplasia. Multiple cycles of systemic PDX-1 RNAi lipoplexes were well tolerated in both immune-deficient and immune-competent mice. bi-shRNA^humanPDX-1^ lipoplexes nearly completely ablated human pancreatic cancer tumor volume and prolonged survival in a in a xenograft mouse of human pancreatic cancer. Bi-shRNA^mousePDX-1^ lipoplexes prevented death from hypoglycemia and hyperinsulinemia in an insulinoma mouse model, demonstrating relief of devastating effects of excessive hormonal secretion in this lethal model of insulinoma. Three cycles of shRNA^mousePDX-1^ lipoplexes were well tolerated and effectively reversed hyperinsulinemia and hypoglycemia in an immune-competent mouse model of islet neoplasia. In these models, murine specific PDX-1 knockdown was associated with mild temporal hyperglycemia due to suppression of *in situ* islet PDX-1 expression and related islet hormones, as well as enhanced islet apoptosis. These data demonstrate effective systemic delivery of the platform to the PDX-1 target and suggest a regenerative capacity of the murine endocrine pancreas. Combined with the overexpression of PDX-1 found in human specimens of pancreatic neuroendocrine tumors and nesidioblastosis, these data demonstrate that PDX-1 is a potential therapeutic target for pancreatic cancer, insulinonoma and islet neoplasia using a novel RNAi effector platform.

## Supporting Information

Figure S1 bi-shRNA^humanPDX-1^ therapies for PANC-1 SCID mice do not affect serum insulin levels, glucose levels, PDX-1 expression or insulin expression in islet cells. Three cycles of treatment with empty vector or bi-shRNA^humanPDX-1^ were applied to PANC-1 xenografted SCID mice. Fasting serum was collected on days 0, 7, 21, 35 and 120 following the initial therapy. At least 5 mice were sacrificed and necropsy was performed on days 0, 43, 90 and 120 after the initial treatment. Insulin levels (a), along with corresponding glucose levels (b) are shown. In each panel (a,b) blue lines present control data while red lines present treatment-group data. Immunostaining for PDX-1 or insulin expression was performed on pancreatic specimens. The image was viewed and photographed under microscopy equipped with a digital camera (c) (×200).
(TIF)

Figure S2 Knockdown of PDX-1 expression affects cell cycle proteins in the pancreas in vivo. Whole pancreata were obtained from empty vector or shRNA^mousePDX-1^ -treated SSTR*1/5* ^−/−^ mice 72 h following administration. Western blot of pancreata lysate with antibodies against PDX-1, Cyclin D, Cyclin E, CdK4 and Cdk2 was performed and analyzed.
(TIF)

Figure S3 shRNA^mousePDX-1^ therapies for βTC-6 SCID mice knockdown PDX-1 expression, inhibit insulin and PP expression, and increase apoptosis of islet cells. Immunostaining for PDX-1, insulin, and PP was performed and TUNEL assay for apoptosis was carried out on pancreatic sections. The image was viewed and photographed under microscopy equipped with a digital camera (×200).
(TIF)

Figure S4 Species specific-bi-shRNA^PDX-1^/shRNA^PDX-1^ therapy prolonged survival of beta TC-6 SCID mice and PANC-1 mice. Comparisons of betaTC-6 SCID mouse survival rates are shown in the following paired groups: (a) three cycles vs control, (b) two cycles vs three cycles, (c) one cycle vs contro and two cycles vs one cycle(d) in shRNA^mousePDX-1^ therapy, (e) three cycles of bi-shRNA^mousePDX-1^ vs control and (f) three cycles of shRNA^mousePDX-1^ vs bi- shRNA^mousePDX-1^. Comparisons of PANC-1 SCID mouse survival rates are shown in the followings: (g) three cycles of bi-shRNA^humanPDX-1^ vs control group and (h) control group and untreated tumor mice.
(TIF)
