# Supplementary figures and images for: PDX-1 Is a Therapeutic Target for Pancreatic Cancer, Insulinoma and Islet Neoplasia Using a Novel RNA Interference Platform
Source: PLoS One. 2012 Aug 8;7(8):e40452. doi: 10.1371/journal.pone.0040452 (PMC3414490; doi:10.1371/journal.pone.0040452)

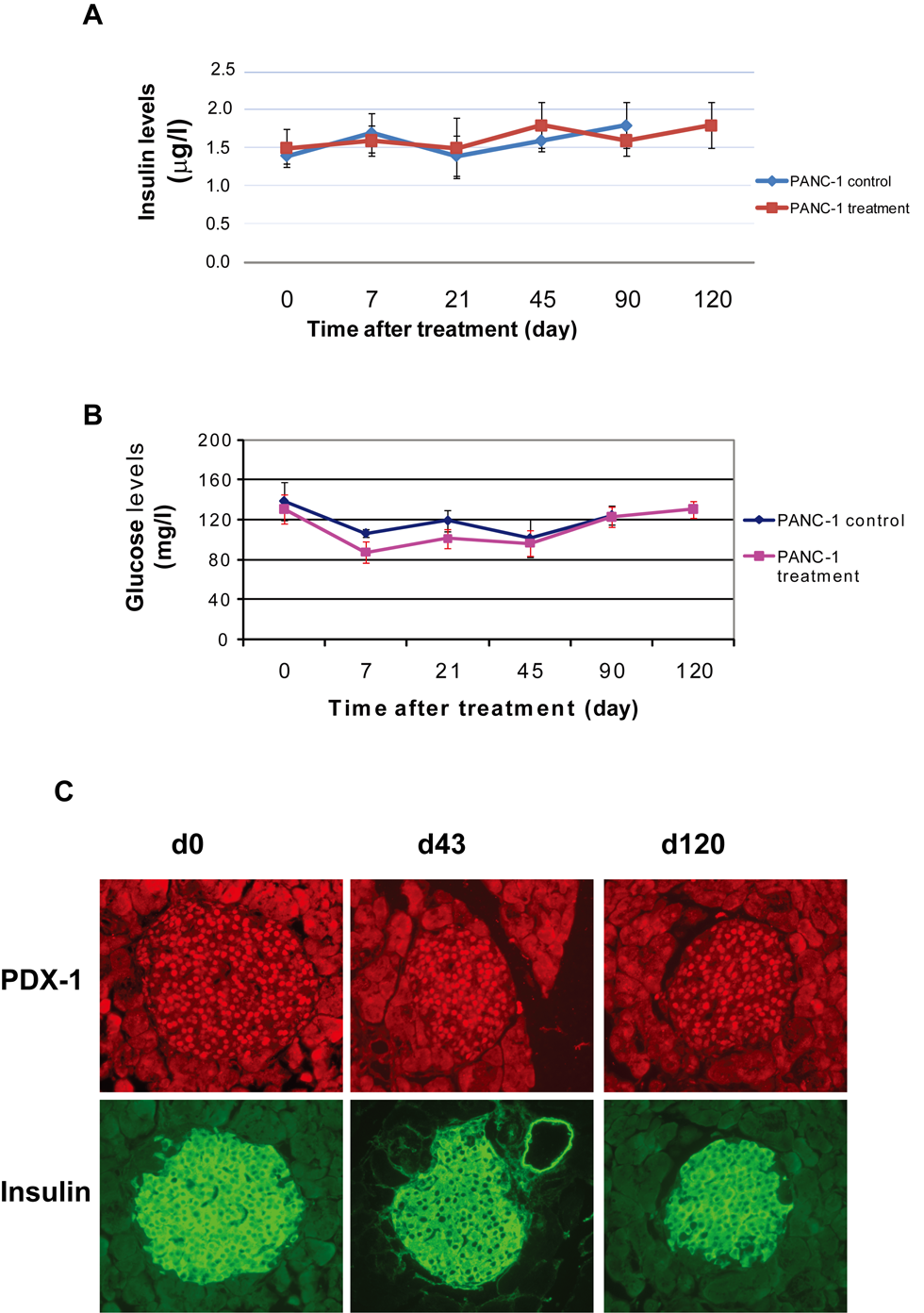

Supplement: Figure S1 — bi-shRNAhumanPDX-1 therapies for PANC-1 SCID mice do not affect serum insulin levels, glucose levels, PDX-1 expression or insulin expression in islet cells. Three cycles of treatment with empty vector or bi-shRNAhumanPDX-1 were applied to PANC-1 xenografted SCID mice. Fasting serum was collected on days 0, 7, 21, 35 and 120 following the initial therapy. At least 5 mice were sacrificed and necropsy was performed on days 0, 43, 90 and 120 after the initial treatment. Insulin levels (a), along with corresponding glucose levels (b) are shown. In each panel (a,b) blue lines present control data while red lines present treatment-group data. Immunostaining for PDX-1 or insulin expression was performed on pancreatic specimens. The image was viewed and photographed under microscopy equipped with a digital camera (c) (×200). (TIF) [file pone.0040452.s001.tif]

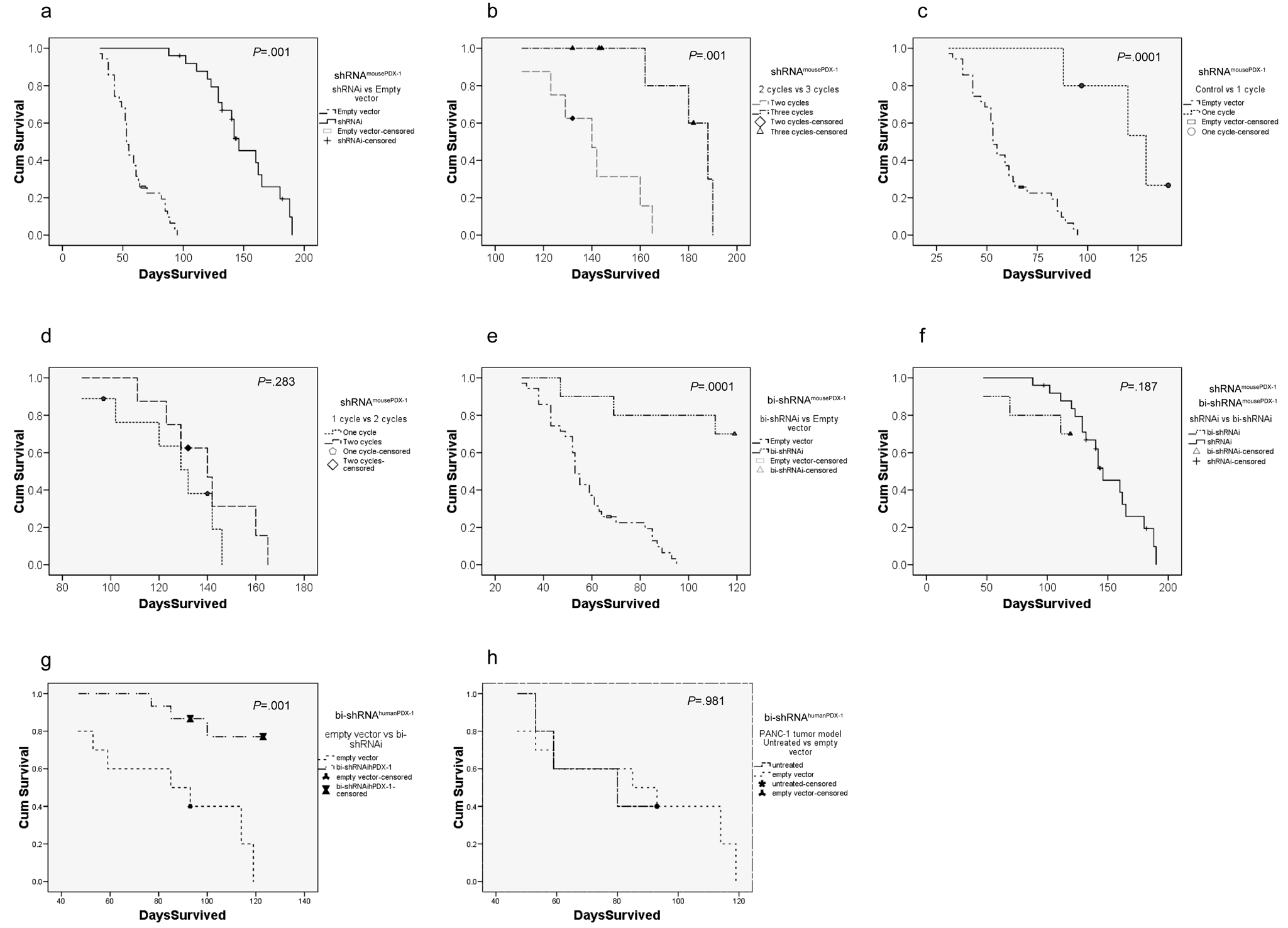

Supplement: Figure S2 — Knockdown of PDX-1 expression affects cell cycle proteins in the pancreas in vivo. Whole pancreata were obtained from empty vector or shRNAmousePDX-1 -treated SSTR1/5 −/− mice 72 h following administration. Western blot of pancreata lysate with antibodies against PDX-1, Cyclin D, Cyclin E, CdK4 and Cdk2 was performed and analyzed. (TIF) [file pone.0040452.s002.tif]

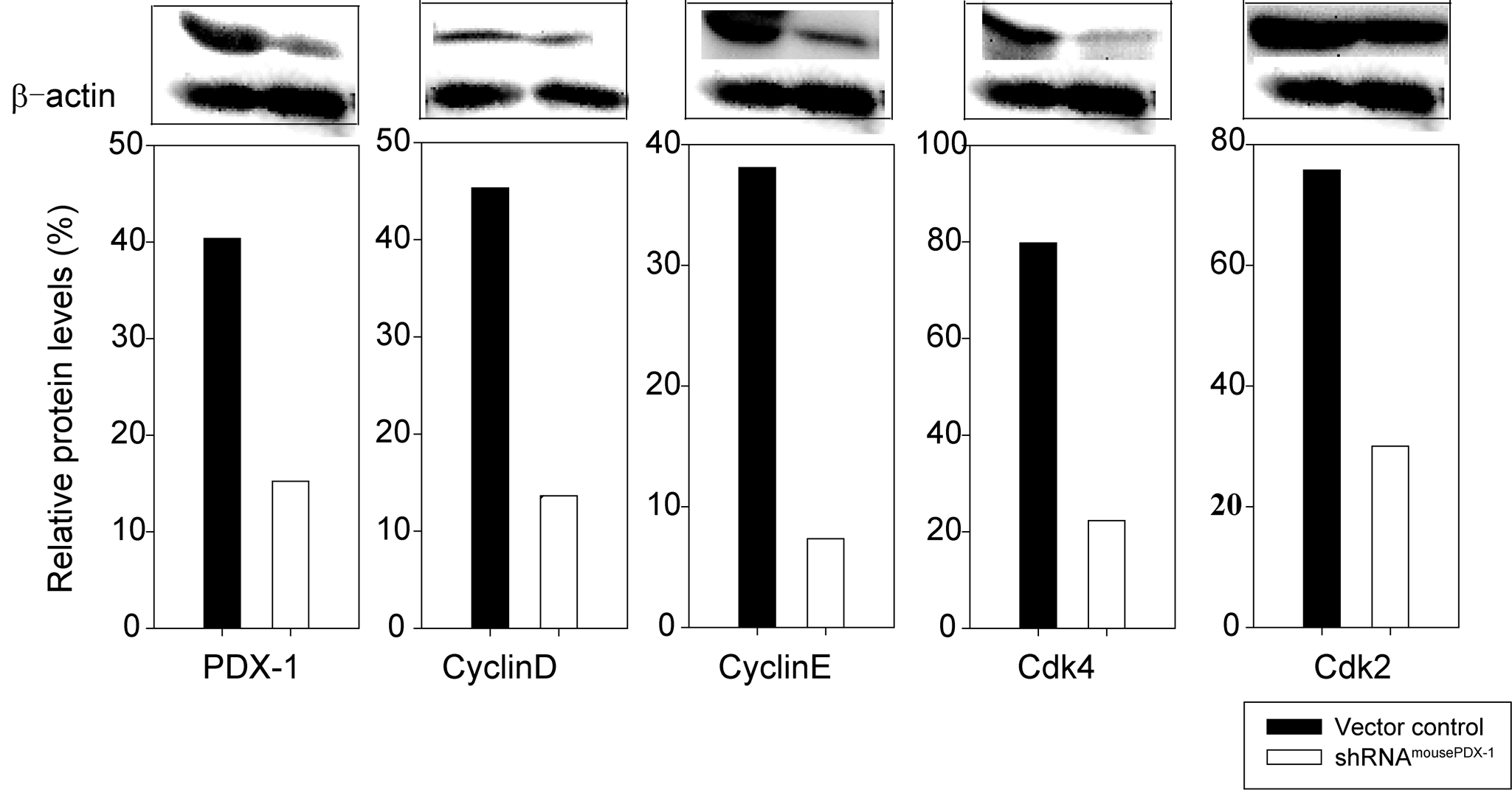

Supplement: Figure S3 — shRNAmousePDX-1 therapies for βTC-6 SCID mice knockdown PDX-1 expression, inhibit insulin and PP expression, and increase apoptosis of islet cells. Immunostaining for PDX-1, insulin, and PP was performed and TUNEL assay for apoptosis was carried out on pancreatic sections. The image was viewed and photographed under microscopy equipped with a digital camera (×200). (TIF) [file pone.0040452.s003.tif]

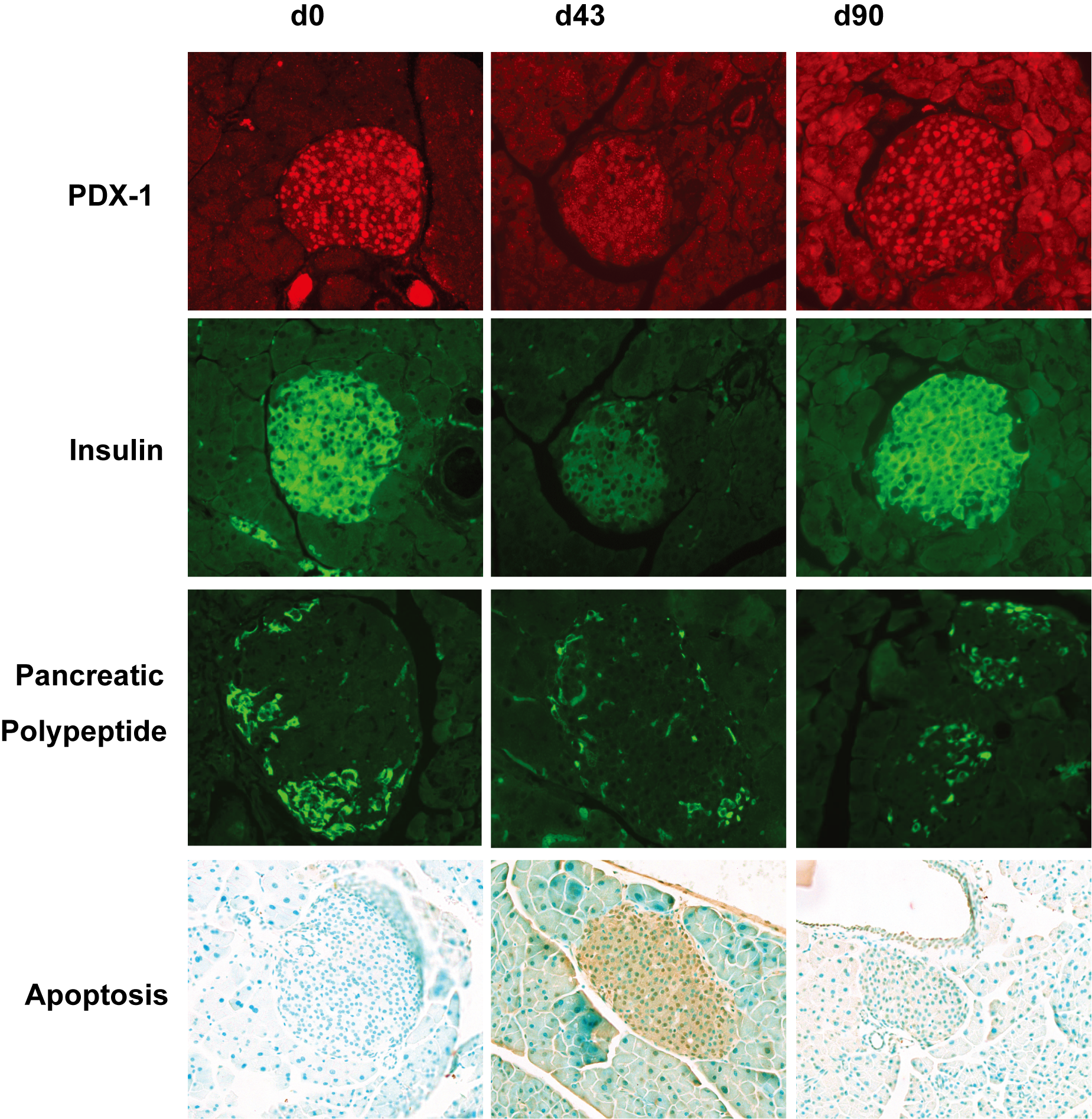

Supplement: Figure S4 — Species specific-bi-shRNAPDX-1/shRNAPDX-1 therapy prolonged survival of beta TC-6 SCID mice and PANC-1 mice. Comparisons of betaTC-6 SCID mouse survival rates are shown in the following paired groups: (a) three cycles vs control, (b) two cycles vs three cycles, (c) one cycle vs contro and two cycles vs one cycle(d) in shRNAmousePDX-1 therapy, (e) three cycles of bi-shRNAmousePDX-1 vs control and (f) three cycles of shRNAmousePDX-1 vs bi- shRNAmousePDX-1. Comparisons of PANC-1 SCID mouse survival rates are shown in the followings: (g) three cycles of bi-shRNAhumanPDX-1 vs control group and (h) control group and untreated tumor mice. (TIF) [file pone.0040452.s004.tif]
